# Changes in thyroid function after thermal ablation of thyroid nodules

**DOI:** 10.3389/fendo.2025.1557725

**Published:** 2025-02-27

**Authors:** Song Li, Ming-an Yu, Zhen-long Zhao, Ying Wei, Li-li Peng, Yan Li

**Affiliations:** China-Japan Friendship Hospital, Beijing, China

**Keywords:** thermal ablation, thyroid nodules, thyroid function, papillary thyroid carcinoma, benign thyroid nodules

## Abstract

**Purpose:**

To evaluate changes in thyroid function post-thermal ablation (TA) of thyroid nodules and to identify risk factors associated with post-ablation thyroid function abnormalities.

**Materials and methods:**

A retrospective analysis of 2,264 cases treated with TA between June 2015 and July 2024 was conducted, including 1,169 benign thyroid nodules (BTNs) and 1,095 papillary thyroid carcinoma (PTC) cases. Thyrotropin (TSH), free triiodothyronine (FT3), and free thyroxine (FT4) levels were measured before treatment and at 1, 3, 6, 9, and 12 months post-ablation.

**Result:**

FT3 levels remained significantly reduced at 12 months post-ablation (3.04 ± 0.42 vs. 3.15 ± 0.36 pg/mL; *p* < 0.001). In contrast, FT4 levels showed a persistent increase at 12 months (1.36 ± 0.69 vs. 1.27 ± 0.15 ng/dL; *p* < 0.001). Although TSH levels decreased slightly over time, they remained elevated at 12 months compared to baseline (1.80 ± 1.17 vs. 1.73 ± 0.84 μIU/mL; *p* = 0.029). At the end of the follow-up period, the incidence of thyroid function abnormalities was 5.07% (18/355), with only one patient requiring Thiamazole for antithyroid therapy. The cumulative incidence of thyroid function abnormalities was notably higher in the PTC group compared to the BTN group (17.80% vs. 10.94%; *p* < 0.001). Pre-ablation TSH levels (OR= 2.06; 95% CI, 1.77–2.39; *p* < 0.001), Hashimoto’s thyroiditis (OR = 2.66; 95% CI, 1.88–3.77; *p* < 0.001), and multiple nodules were positively correlated with the occurrence of thyroid function abnormalities. The cutoff value of TSH was 2.015 μIU/mL with a sensitivity of 0.527 and a specificity of 0.246 (AUC = 0.625).

**Conclusion:**

Thermal ablation had a minimal impact on thyroid function. Pre-ablation TSH levels, Hashimoto’s thyroiditis, and multiple nodules were risk factors for post-ablation thyroid function abnormalities.

## 1 Introduction

Thyroid nodules are a prevalent clinical finding, with their detection rate steadily rising due to increased diagnostic imaging and aging populations (1–4). For symptomatic benign nodules or confirmed malignant nodules, hemithyroidectomy and thyroidectomy remain standard and effective treatment (5–7). Despite their effectiveness, these procedures have inherent risks that can impact patient quality of life (8, 9). In one large study, approximately 29% of patients developed hypothyroidism post-hemithyroidectomy, with 34% experiencing transient hypothyroidism and 23% requiring long-term hormone replacement (10).

In recent years, minimally invasive thermal ablation (TA) has emerged as an alternative treatment for thyroid nodules. Techniques such as microwave ablation (MWA) and radiofrequency ablation (RFA) have demonstrated efficacy in reducing nodule volume, preserving thyroid function, and minimizing complications compared to surgery (11–14). Current guidelines endorse TA for managing BTNs and select PTC cases, particularly for patients ineligible for or unwilling to undergo surgery. However, thyroid function abnormalities, such as hypothyroidism or subclinical hypothyroidism, have been observed in a few patients post-ablation, emphasizing the need for regular thyroid function monitoring (15–17).

Existing studies evaluating the impact of TA on thyroid function are limited by small sample sizes, heterogeneous patient populations, and inconsistent findings. Furthermore, risk factors contributing to thyroid function abnormalities following ablation remain underexplored. To address these gaps, this large-scale retrospective study analyzed 2,264 patients undergoing TA, systematically evaluating changes in thyroid function over a 12-month period and trying to identify independent predictors of post-ablation function abnormalities. These findings aim to guide clinical follow-up strategies and optimize patient outcomes.

## 2 Materials and methods

This retrospective study was approved by the Institutional Review Board of the China-Japan Friendship Hospital. Written informed consent was obtained from all patients prior to undergoing thermal ablation, which included consent for the anonymous publication of examination results and imaging data.

### 2.1 Patient

**Figure 1:**
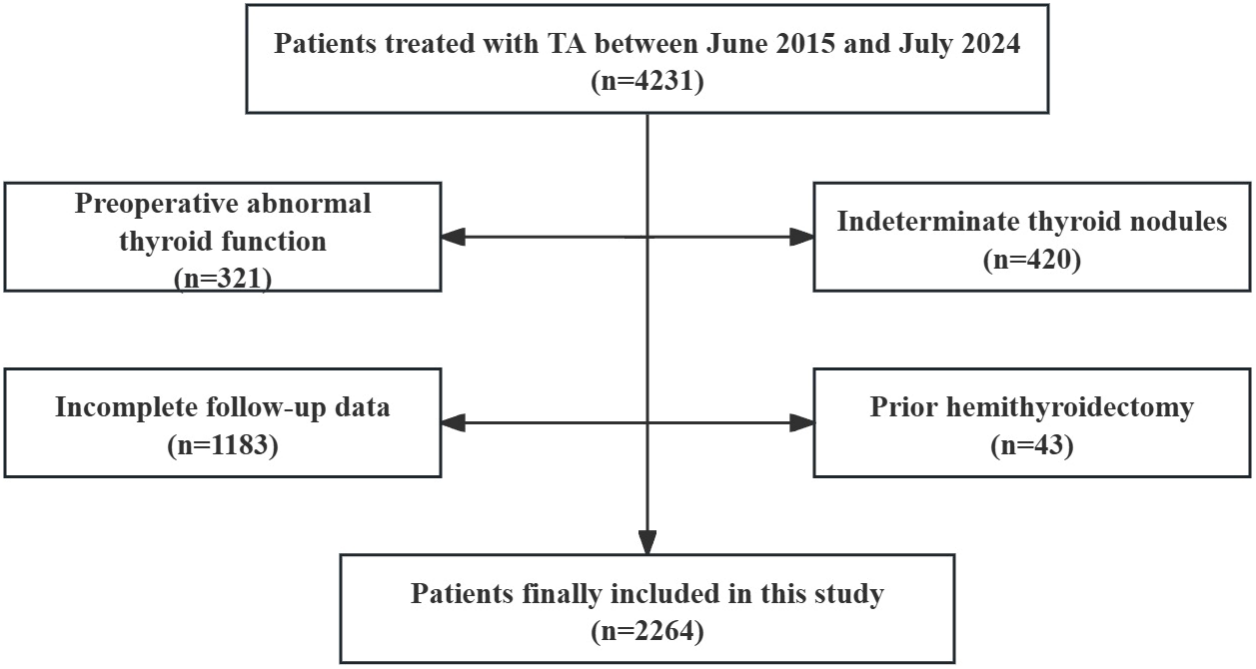
Study flowchart.

Clinical data of patients with thyroid nodules who underwent TA between June 2015 and July 2024 were retrospectively analyzed. The inclusion criteria were as follows: (i) a diagnosis of benign thyroid nodule (BTN) or papillary thyroid cancer (PTC) confirmed by ultrasound-guided fine-needle aspiration biopsy (FNA), (ii) patients were ineligible for surgery due to a high risk of general anesthesia and tracheal intubation, and (iii) a follow-up time of at least one month. The exclusion criteria were as follows: (i) a PTC tumor stage greater than T2N0M0, (ii) abnormal thyroid function before ablation, (iii) antithyroid drug or hormone replacement drug use before ablation; (iv) incomplete follow-up data; and (v) prior hemithyroidectomy (18) (**Figure 1**).

### 2.2 Preablation assessment

A Logiq E9 ultrasound scanner (GE Healthcare, US) with a 9.0 MHz linear probe was used for the assessment. The three orthogonal diameters (the largest diameter and two perpendicular diameters) and the location of the nodules were measured and recorded. Each measurement was performed by three physicians, and the average was recorded as the final result (18).

The normal reference ranges for thyroid function parameters used in this study are as follows: Triiodothyronine (T3) at 0.8–2.0 ng/mL, Free triiodothyronine (FT3) at 2.0–4.4 pg/mL, Thyroxine (T4) at 5.1–14.1 μg/dL, Free thyroxine (FT4) at 0.93–1.7 ng/dL, and Thyrotropin (TSH) at 0.27–4.20 μIU/mL. Antibodies against thyroid peroxidase (TPOAb) at 0–34 IU/mL, Antibodies against thyroglobulin (TGAb) at 0–115 IU/mL. The diagnostic criteria for Hashimoto’s thyroiditis include positive thyroid autoantibodies (TPOAb, TgAb) and ultrasound findings of diffusely hypoechoic thyroid tissue.

### 2.3 TA procedure

TA was performed by two radiologists with more than 5 years of experience. Before ablation, contrast-enhanced ultrasound (CEUS) was performed to observe the enhancement mode and margin of the nodule. The MWA (Intelligent Basic Type Microwave Tumor Ablation System, Nanjing ECO Microwave System) and RFA (Cooltip Radiofrequency Ablation System [Covidien]) were performed under local anesthetic conditions. After ablation, CEUS was repeated to evaluate the ablation effect. Complete ablation was defined as a nonenhanced ablation zone completely covering the benign tumor and extending at least 2 mm from the original PTC margin on CEUS. At the end of the procedure, the puncture site was compressed for 30 minutes, and the patient remained under observation for 2 hours to monitor potential complications (18).

### 2.4 Follow-up visits

At the clinical follow-up, various examinations, including ultrasound and thyroid function tests, were performed at 1, 3, 6, 9, and 12 months, and every 6 months thereafter. Thyroid function abnormalities were defined as one or more abnormalities post-ablation, including hyperthyroidism, subclinical hyperthyroidism, hypothyroidism, and subclinical hypothyroidism. Subclinical hyperthyroidism was diagnosed when TSH was lower than the normal range but (f)T3 and (f)T4 were normal, and subclinical hypothyroidism was diagnosed when TSH was higher than the normal range but (f)T3 and (f)T4 were normal (18).

### 2.5 Statistical analysis

Statistical analysis was performed using R software (version 4.2.2) and MSTATA software (www.mstata.com). Data conforming to a normal distribution were expressed as mean ± standard deviation (SD), while non-normally distributed data were expressed as median and interquartile range (25th-75th percentile, IQR). For data fitting a normal distribution, a two-sided Student's t-test was applied. For non-normally distributed data, independent two-sided Mann-Whitney U tests and Wilcoxon signed-rank tests were employed to compare medians of continuous variables. Chi-square tests and Fisher's exact tests were used for categorical data. To analyze risk factors for thyroid function abnormalities from 1 to 12 months post-ablation, one-way binary logistic regression was initially conducted, followed by multivariable logistic regression for significant variables. Differences were considered statistically significant when P < 0.05.

## 3 Result

The baseline characteristics of the 2,264 cases analyzed in present study are summarized in **Table 1**. The median age of participants was 44 years (IQR: 35–55 years), with 72.3% being female. Of the nodules treated, 51.6% were classified as BTN and 48.4% as PTC. Most nodules were located in the right lobe (48.9%), followed by the left lobe (47.3%) and the isthmus (3.8%). The median maximum nodule diameter was 1.10 cm (IQR: 0.70–2.90 cm). Hashimoto’s thyroiditis was present in 17.6% of patients, and BRAF V600E mutations were identified in 43.3% of cases. Solid nodules comprised 84.3% of the cases, while 15.7% were mixed cystic-solid nodules.

**Table 1:** Cases demographics and baseline characteristics.

Characteristic	N = 2,264
**Age (years)**	44 (35, 55)
Gender	
Male	627 (27.7%)
Female	1,637 (72.3%)
Nodules type	
Benign thyroid nodules	1,169 (51.6%)
Papillary thyroid carcinoma	1,095 (48.4%)
Location of the Nodules	
Left lobe	1,070 (47.3%)
Right lobe	1,108 (48.9%)
Isthmus	86 (3.8%)
**Maximum diameter (cm)**	1.10 (0.70, 2.90)
Hashimoto’s thyroiditis	
None	1,865 (82.4%)
Yes	399 (17.6%)
BRAF V600E mutation	
Negative	1,284 (56.7%)
Positive	980 (43.3%)
Calcification	
None	1,858 (82.1%)
Yes	406 (17.9%)
Composition	
Solid	1,908 (84.3%)
Mixed cystic-solid	356 (15.7%)
Margin	
Smooth	1,059 (46.8%)
Irregular	1,205 (53.2%)
CEUS	
Hypo-enhancement	695 (30.7%)
Iso-enhancement	240 (10.6%)
Hyper-enhancement	1,329 (58.7%)
Technique of TA	
Radiofrequency ablation	471 (20.8%)
Microwave ablation	1,793 (79.2%)
Number of Nodules treated with TA	
1	1,353 (59.8%)
2	570 (25.2%)
3	341 (15.1%)

### 3.1 Changes in thyroid function after thermal ablation

Baseline thyroid function values were recorded as follows: FT3 (3.15 ± 0.36 pg/mL), FT4 (1.27 ± 0.15 ng/dL), and TSH (1.73 ± 0.84 μIU/mL).

**Figure 2:**
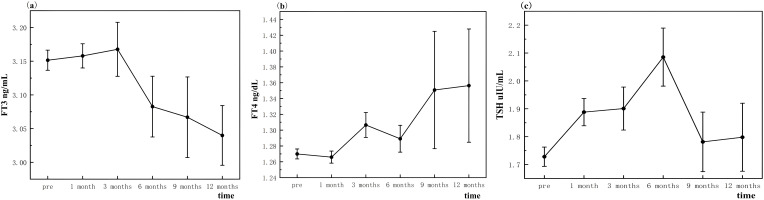
Changes in thyroid function after thermal ablation.

Serum FT3 levels demonstrated a consistent decline at 6, 9, and 12 months following thermal ablation, with mean values significantly lower than pre-ablation levels (3.08 ± 0.60, 3.07 ± 0.59, and 3.04 ± 0.42 vs. 3.15 ± 0.36 pg/mL, respectively; *p* < 0.001), this reduction suggests a persistent suppression of triiodothyronine production or release, likely due to thermal effects on thyroid parenchyma (**Figure 2A**).

In contrast, serum FT4 levels exhibited a significant increase at 3, 9, and 12 months post-ablation compared to baseline (1.31 ± 0.27, 1.35 ± 0.74, and 1.36 ± 0.69 ng/dL vs. 1.27 ± 0.15 ng/dL, respectively; *p* < 0.05). This elevation may reflect compensatory mechanisms by the thyroid gland to maintain metabolic stability (**Figure 2B**).

TSH levels showed an initial significant rise at 1 month (1.89 ± 1.14 μIU/mL; *p* < 0.05), followed by sustained elevations at 3, 6, and 12 months post-ablation (1.90 ± 1.33, 2.09 ± 1.40, and 1.80 ± 1.17 vs. 1.73 ± 0.84 μIU/mL, respectively; *p* < 0.05). These findings indicate a feedback response to alterations in thyroid hormone levels, possibly due to reduced thyroid reserve (**Figure 2C**).

### 3.2 Incidence of thyroid function abnormality after thermal ablation

At 1 month post-ablation, thyroid function abnormalities were detected in 7.42% (153/2,061) of patients. Among these, subclinical hypothyroidism (3.59%) was the most frequent, followed by subclinical hyperthyroidism (2.66%), hyperthyroidism (0.82%), and hypothyroidism (0.33%). The overall incidence peaked at 3 months, rising to 11.33% (130/1,147). At this time point, hyperthyroidism (3.40%) became the most common abnormality, while subclinical hypothyroidism (4.27%) and subclinical hyperthyroidism (3.05%) were also prevalent. Hypothyroidism remained relatively rare (0.61%).

From 6 months onwards, the incidence of thyroid function abnormalities showed a declining trend. At 6 months, the overall rate decreased to 10.08% (70/694), with subclinical hypothyroidism remaining the most common abnormality (6.05%). At 9 months, the incidence further dropped to 6.31% (24/380), and by 12 months, only 5.07% (18/355) of patients exhibited any form of thyroid function abnormality. At this time point, subclinical hypothyroidism (3.66%) continued to dominate, while hyperthyroidism was observed in just 0.28%, and no cases of hypothyroidism were recorded. (**Table 2**)

**Table 2:** Incidence of thyroid function abnormality after thermal ablation.

Follow-up time	Hyperthyroidism	Hypothyroidism	Subclinical hyperthyroidism	Subclinical hypothyroidism	Total
1 month (n=2061)	17 (0.82%)	7 (0.33%)	55 (2.66%)	74 (3.59%)	153 (7.42%)
3 months (n=1147)	39 (3.40%)	7 (0.61%)	35 (3.05%)	49 (4.27%)	130 (11.33%)
6 months (n=694)	11 (1.58%)	6 (0.86%)	11 (1.58%)	42 (6.05%)	70 (10.08%)
9 months (n=380)	7 (1.84%)	1 (0.26%)	7 (1.84%)	9 (2.36%)	24 (6.31%)
12 months (n=355)	1 (0.28%)	0	4 (1.12%)	13 (3.66%)	18 (5.07%)

Patients who developed overt hypothyroidism showed a median TSH of 7.58 μIU/mL, FT3 of 2.78 pg/mL, and FT4 of 0.88 ng/dL. Subclinical hypothyroidism patients had a median TSH of 5.01 μIU/mL, FT3 of 3.13 pg/mL, and FT4 of 1.19 ng/dL. Conversely, patients who developed hyperthyroidism exhibited a median TSH of 0.025 μIU/mL, FT3 of 4.53 pg/mL, and FT4 of 2.02 ng/dL. Subclinical hyperthyroidism patients had a median TSH of 0.119 μIU/mL, FT3 of 3.425 pg/mL, and FT4 of 1.44 ng/dL.

By the end of the follow-up period, 17 patients exhibited thyroid function abnormalities, including 1 case of hyperthyroidism, 4 cases of subclinical hyperthyroidism, and 13 cases of subclinical hypothyroidism. Notably, only one patient with Hashimoto's thyroiditis required Thiamazole for antithyroid therapy, with subsequent hormone levels remaining stable during follow-up.

### 3.3 Changes in thyroid function among patients with benign thyroid nodules and papillary thyroid carcinoma treated with thermal ablation

The incidence of thyroid function abnormalities differed between BTNs and PTC cases. At 1 month post-ablation, the rates of functional abnormalities were similar between the BTN (7.59%, 79/1040) and PTC (7.24%, 74/1021 ) groups (*p* = 0.763). However, by 3 months, the PTC group showed a significantly higher incidence compared to the BTN group (14.01% vs. 7.35%; *p* < 0.001). This trend persisted at 6 months (12.36% vs. 7.32%; *p* = 0.028), there were no significant differences at 9 or 12 months.

The cumulative incidence of thyroid function abnormalities was notably higher in the PTC group (17.80%) compared to the BTN group (10.94%; *p* < 0.001). These results are summarized in **Table 3**.

**Table 3:** Changes in thyroid function among cases with BTNs and PTC treated with thermal ablation.

Follow-up time	BTNs	PTC	*P*
1-month	7.59% (79/1040)	7.24% (74/1021)	0.763
3-month	7.35% (34/462)	14.01% (96/685)	<0.001*
6-month	7.32% (23/314)	12.36% (47/380)	0.028*
9-month	6.15% (8/130)	6.40% (16/250)	0.925
12-month	6.74% (11/163)	3.60% (7/194)	0.177
Total	10.94% (128/1169)	17.80% (195/1095)	<0.001*

* mean statistically significant difference (p < 0.05).

### 3.4 Risk factors analysis of thyroid function abnormality

**Figure 3:**
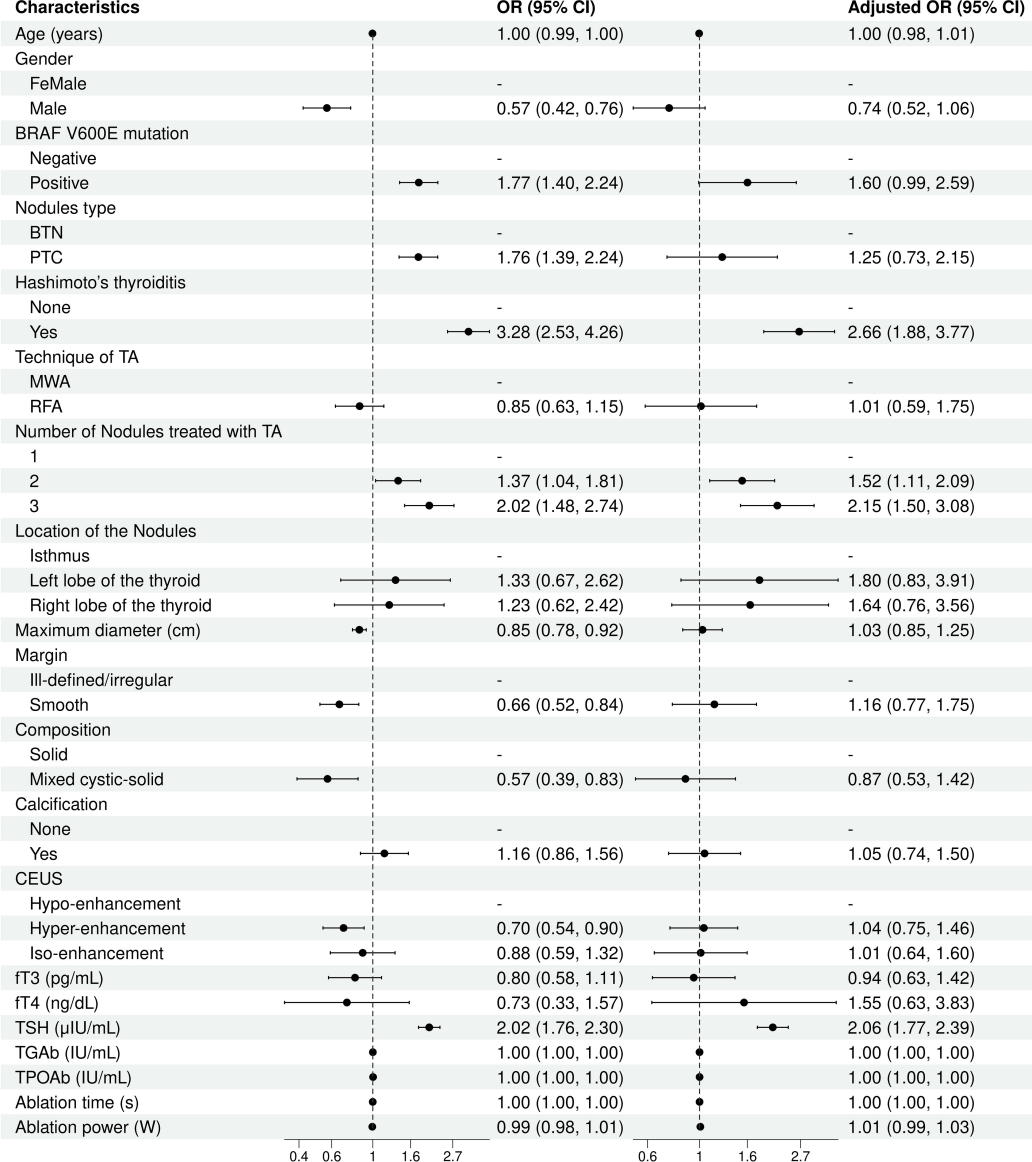
Risk factors analysis of thyroid function abnormality.

Logistic regression analyses identified significant independent risk factors for thyroid function abnormalities post-ablation. Pre-ablation TSH levels were associated with an increased risk of thyroid function abnormality (OR= 2.06; 95% CI, 1.77–2.39; *p* < 0.001). The cutoff value of TSH was 2.015 μIU/mL with a sensitivity of 0.527 and a specificity of 0.246 (AUC = 0.625). The presence of Hashimoto’s thyroiditis was another strong predictor (OR = 2.66; 95% CI, 1.88–3.77; *p* < 0.001). Additionally, treatment involving multiple nodules increased the likelihood of thyroid function abnormalities, with higher risks observed for two nodules (OR = 1.52; 95% CI, 1.11–2.09; *p* = 0.010) and three nodules (OR = 2.15; 95% CI, 1.50–3.08; *p* < 0.001). Other factors, such as age, gender, and BRAF V600E mutation, were not significant in the multivariate analysis (**Figure 3**).

### 3.5 Complications

The complication rate was 2.12% (48/2264), with 48 patients experiencing transient hoarseness, which resolved spontaneously within 6 months without treatment. No other complications were observed.

## 4 Discussion

This study demonstrates that TA may temporarily impact thyroid function, particularly in cases involving PTC. By systematically analyzing a large cohort, we identified key risk factors for post-ablation thyroid function abnormalities, including elevated pre-ablation TSH levels, the presence of Hashimoto’s thyroiditis, and treatment involving multiple nodules. These findings provide valuable insights into patient selection and post-procedural management strategies, highlighting the need for tailored approaches to minimize the risk of function abnormalities following ablation.

At 12 months post-ablation, thyroid function abnormalities was 5.07% (18/355). The observed alterations in thyroid function likely result from direct thermal effects on the thyroid parenchyma and associated vascular changes. Elevated TSH levels at 1, 3, and 6 months post-ablation reflect a feedback response to reduced thyroid hormone synthesis, indicative of transient glandular impairment. Meanwhile, the sustained increase in FT4 levels at later stages suggests compensatory mechanisms from surviving thyroid tissue, highlighting the thyroid gland's adaptive capacity (19).

While the majority of patients showed a reduction in thyroid function post-ablation, a subset experienced transient thyroid hyperfunction. In our study, we observed that 74 (98.67%, 74/75 ) of the patients developed temporary hyperthyroidism, only one patient with Hashimoto’s thyroiditis exhibited hyperthyroidism at 12 months post-ablation. This phenomenon may be attributed to thyroiditis or inflammatory responses following thermal ablation, which can sometimes cause a temporary release of thyroid hormones. Previous studies have also documented transient hyperthyroidism as a known complication of both microwave and radiofrequency ablation, with the condition typically resolving within a few months as the inflammatory response subsides (19). The persistent hyperthyroidism in this patient may have resulted from thermal ablation exacerbating the inflammatory process of thyroiditis, causing the patient to develop hyperthyroidism despite having normal thyroid function before ablation. Though one patients required specific interventions, hyperthyroidism may occasionally be managed with beta-blockers and Thionamides for symptom control, as suggested in existing literature (20).

These findings suggest that thyroid function abnormalities post-thermal ablation are generally transient and predominantly subclinical, with most cases resolving within 12 months, with spontaneous resolution without specific treatment. The results highlight the need for routine thyroid function monitoring, particularly within the first three months post-ablation, to ensure timely detection and management of abnormalities.

In our study, the complication rate was relatively low, with 2.12% of patients experiencing adverse effects. The most common complications were transient hoarseness, which resolved without the need for medical intervention. This aligns with previous reports, which attribute hoarseness to temporary injury to the recurrent laryngeal nerve during ablation procedures, though permanent injury is rare (21). No serious or permanent complications occurred in our cohort. These findings support the overall safety profile of thermal ablation for thyroid nodules and highlight the typically benign nature of the complications associated with the procedure.

The differential impact between BTNs and PTC cases underscores the importance of tailored ablation protocols. PTC patients exhibited higher rates of thyroid function abnormalities at 3 and 6 months. At 3 months post-ablation, 1.08% (5/462) of the BTN group and 5.98% (41/685) of the PTC group thyroid function abnormalities. At 6 months, 0.96% (3/314) of the BTN group and 3.68% (14/380) of the PTC group thyroid function abnormalities. This difference is likely due to the more aggressive ablation required for malignant lesions, leading to greater parenchymal disruption. These findings emphasize the need for personalized follow-up strategies, with more frequent thyroid function monitoring in high-risk populations to enable early intervention.

Risk factor analysis revealed pre-ablation TSH levels as a significant predictor of function abnormalities, potentially indicating pre-existing thyroid stress. Similarly, Hashimoto’s thyroiditis, characterized by chronic lymphocytic infiltration and tissue damage, further predisposed patients to post-procedural abnormalities. Treatment involving multiple nodules also increased function abnormalities risk, likely due to the larger volume of thyroid tissue ablated, reducing overall hormone reserve. These findings underscore the necessity of careful patient assessment and individualized treatment planning.

This study advances existing knowledge by providing a comprehensive analysis of thyroid function changes and their predictors after TA. Compared to previous studies with smaller sample sizes or limited follow-up, our findings offer a clearer understanding of the factors influencing post-ablation outcomes. These insights can inform clinical guidelines and improve patient care.

Despite its strengths, this study has limitations. Its retrospective design may introduce selection bias, and the follow-up period was limited to 12 months, precluding an assessment of long-term thyroid function changes. Additionally, we did not investigate the potential impact of procedural variations, such as ablation technique or energy parameters, on thyroid function. Future prospective studies with extended follow-up and standardized protocols are warranted to validate these findings and explore strategies to mitigate thermal damage.

In conclusion, TA is a minimally invasive and effective treatment for thyroid nodules but may affect thyroid function, particularly in high-risk populations. By identifying key predictors of function abnormalities, this study provides actionable insights to optimize patient selection, customize follow-up protocols, and enhance clinical outcomes. Further research is needed to evaluate the long-term effects of TA and refine procedural techniques to minimize thyroid function abnormalities while maximizing treatment efficacy.

## Data Availability

The dataset analyzed in this study is not publicly available due to restrictions related to patient confidentiality and ethical considerations. Access to the data is restricted to authorized researchers affiliated with the study and is governed by the ethical approval obtained from the Institutional Review Board of the China-Japan Friendship Hospital. Requests for access to the data can be made to the corresponding author, subject to review and compliance with data protection regulations and institutional policies. Requests to access the datasets should be directed to SL, 2475889420@qq.com.
